# Survey of ophthalmologists-in-training in Eastern, Central and Southern Africa: A regional focus on ophthalmic surgical education

**DOI:** 10.12688/wellcomeopenres.15580.1

**Published:** 2019-11-27

**Authors:** William Dean, Stephen Gichuhi, John Buchan, Ibrahim Matende, Ronnie Graham, Min Kim, Simon Arunga, William Makupa, Colin Cook, Linda Visser, Matthew Burton

**Affiliations:** 1 International Centre for Eye Health (ICEH), Clinical Research Department Faculty of Infectious and Tropical Diseases, London School of Hygiene and Tropical Medicine, London, WC1E7HT, UK; 2 Department of Ophthalmology, University of Cape Town, Cape Town, South Africa; 3 Department of Ophthalmology, University of Nairobi, Nairobi, Kenya; 4 College of Ophthalmology of Eastern Central & Southern Africa, Nairobi, Kenya; 5 International Agency for the Prevention of Blindness, Durban, South Africa; 6 Tropical Epidemiology Group, Faculty of Infectious Disease Epidemiology, London School of Hygiene & Tropical Medicine, London, UK; 7 Kilimanjaro Christian Medical Centre, Moshi, Tanzania; 8 Department of Ophthalmology, University of KwaZulu-Natal, Durban, South Africa; 9 Moorfields Eye Hospital NHS Foundation Trust, London, UK

**Keywords:** Ophthalmology, Training, Africa

## Abstract

**Background: **There are 2.7 ophthalmologists per million population in sub-Saharan Africa, and a need to train more. We sought to analyse current surgical training practice and experience of ophthalmologists to inform planning of training in Eastern, Central and Southern Africa.

**Methods:** This was a cross-sectional survey. Potential participants included all current trainee and recent graduate ophthalmologists in the Eastern, Central and Southern African region. A link to a web-based questionnaire was sent to all heads of eye departments and training programme directors of ophthalmology training institutions in Eastern, Central and Southern Africa, who forwarded to all their trainees and recent graduates. Main outcome measures were quantitative and qualitative survey responses.

**Results: **Responses were obtained from 124 (52%) trainees in the region. Overall level of satisfaction with ophthalmology training programmes was rated as ‘somewhat satisfied’ or ‘very satisfied’ by 72%. Most frequent intended career choice was general ophthalmology, with >75% planning to work in their home country post-graduation. A quarter stated a desire to mainly work in private practice. Only 28% of junior (first and second year) trainees felt surgically confident in manual small incision cataract surgery (SICS); this increased to 84% among senior trainees and recent graduates. The median number of cataract surgeries performed by junior trainees was zero. 57% of senior trainees were confident in performing an anterior vitrectomy. Only 29% of senior trainees and 64% of recent graduates were confident in trabeculectomy. The mean number of cataract procedures performed by senior trainees was 84 SICS (median 58) and 101 phacoemulsification (median 0).

**Conclusion:** Satisfaction with post-graduate ophthalmology training in the region was fair. Most junior trainees experience limited cataract surgical training in the first two years. Focused efforts on certain aspects of surgical education should be made to ensure adequate opportunities are offered earlier on in ophthalmology training.

## Introduction

The 49 countries of sub-Saharan Africa (SSA) are home to 12% of the global population, and 23% of the global burden of disease ^1^. The age-standardized prevalence of blindness (presenting visual acuity <3/60) is 1.3%, and 35% of blindness is due to cataract ^2^. Uncorrected refractive error (URE) accounts for 13.2% of blindness, macular degeneration 6.3%, trachoma 5.2%, glaucoma 4.4%, and diabetic retinopathy 2.8% ^3^. URE is the most common identified cause of moderate/severe vision impairment (MSVI) (45.0%) followed by cataract (17.7%) ^3^.

Of the more than 200,000 ophthalmologists worldwide, there are only 2,700 in SSA; a ratio of 2.7 ophthalmologists per million population ^4^. This compares to 27,000 ophthalmologists for the 323 million people of the United States, a ratio of 83 per million ^4, 5^.

In absolute numbers, 1.7 million people in SSA are blind from cataract, and a further 3.1 million have MSVI. The cataract surgery rate needed to eliminate cataract visual impairment at the level of 6/18 has been estimated from mathematical modelling to range from 1,200 to 4,500 surgeries/year/million population in different communities within SSA ^6^. Thus, with an average of 2.7 ophthalmologists per million population in SSA, each ophthalmologist would have to perform a mean of 444–1667 cataract operations annually. To deal with the backlog of 4.8 million people with cataract blindness and MSVI, each individual ophthalmologist would have to perform over 3,500 cataract operations, in addition to the numbers required to tackle incident cataract.

A number of ophthalmology trainee surveys have been conducted over the past ten years, including Nigeria ^7^, the USA ^8, 9^, Canada ^10^, Jordan ^11^, India ^12^, and China ^13^, but no international survey of the training programs in Eastern, Central, and Southern Africa has been published to understand the current training provision and experience as a whole.

Within Eastern, Central, and Southern Africa there are 24 training institutions ( Table 1). In total, 21 are Anglophone, two are Lusophone, and one is Francophone. In South Africa, the College of Ophthalmology provides a national standard curriculum. In 2013, OSEA (Ophthalmology Society of Eastern Africa) and EACO (Eastern Africa College of Ophthalmologists) merged to form COECSA (College of Ophthalmologists of Eastern, Central and Southern Africa). Many training institutions within COECSA have started using a collaboratively developed, standardised curriculum ^14^.

**Table 1.** Ophthalmology training institutions in Eastern, Central and Southern Africa region ^16^.

Country	Institution	Duration (years)	Degree	Number of faculty	Yearly capacity	No. of responses
**Angola**	IONA Eye Institute, Luanda	4	Ophthalmology specialist	4	4	0
**Ethiopia**	Addis Ababa University, Medical Faculty, Dept. of Ophthalmology	4	Specialty certificate	11	7	0
**Ethiopia**	Jimma University	4	Specialty certificate	6	6	0
**Ethiopia**	University of Gondar	4	Specialty certificate	7	6	10
**Kenya**	University of Nairobi, College of Health Sciences, School of Medicine	3	MMed (Ophth)	20	12	34
**Madagascar**	Faculty of Medicine, Antananarivo	4	Degree	4	10	0
**Malawi**	College of Medicine, University of Malawi, Blantyre	4	MMed	4	2	1
**Mozambique**	Maputo Central Hospital	4	Ophthalmology specialist	5	4	0
**Rwanda**	Rwanda Institute of Ophthalmology, Kigali	4	MMed (Ophth)	3	4	0
**South Africa**	Stellenbosch University	4	MMed & FCOphth	6	2	6
**South Africa**	University of the Free State, Bloemfontein	4.5	MMed & FCOphth	2	2	1
**South Africa**	University of Cape Town	4	MMed & FCOphth	17	3	7
**South Africa**	University of KwaZulu- Natal, Durban	4	MMed & FCOphth	13	3	4
**South Africa**	Sefako Makgatho Health Sciences University	4	MMed & FCOphth	5	2	0
**South Africa**	University of Pretoria	4	MMed & FCOphth	5	2	0
**South Africa**	Walter Sisulu University Umtata	4	MMed & FCOphth	4	2	1
**South Africa**	Wits University Johannesburg	4	MMed & FCOphth	16	4	6
**Tanzania**	Kilimanjaro Christian Medical University College, Arusha	4	MMed (Ophth)	9	10	10
**Tanzania**	Muhimbili University of Health and Allied Sciences, Dar es Salaam	4	MMed (Ophth)	6	5	0
**Uganda**	Makerere University, College of Health Sciences, Kampala	3	MMed (Ophth)	4	8	16
**Uganda**	Mbarara University of Science and Technology	3	MMed (Ophth)	6	8	9
**Zambia**	University of Zambia School of Medicine Lusaka	4	MMed (Ophth)	4	4	3
**Zimbabwe**	University of Zimbabwe, Dept. of Ophthalmology, Harare	4	Mastersin Medicine	5	6	14
**Zimbabwe**	United Bulawayo Hospitals	4	MMEd & FRCOphth	2	3	0

There is substantial variability in ophthalmology training between countries in terms of numbers of trainees enrolled relative to the population size, available faculty, facilities, infrastructure, curricula, funding, clinical case volume, materials and equipment. Moreover, anecdotally there seems to be substantial variation in the exposure to surgical training.

In view of recent efforts towards sub-regional harmonization of curricula, increases in enrolment numbers, and adoption of newer educational methods (including competency-based medical education (CBME) and simulation-based surgical education), we conducted a mixed qualitative and quantitative study to survey the objective and subjective perspectives of current and recent ophthalmology trainees in SSA.

## Methods

### Ethics

This study was approved by the research ethics committees of the London School of Hygiene & Tropical Medicine (11795) and the University of Cape Town (259/2017). Participants were provided with information about the nature of the survey prior to participation. As the survey was anonymous, individual written consent was not required prior to voluntary participation, and this was approved by ethics committees. Anonymity was assured by password protection of the survey account, and no personal information being exported into data sheets.

### Study design

A mixed methods research approach was used. Although quantitative-dominant, qualitative data was important in the study of complex interactions underlying ophthalmic surgical training.

A standardized questionnaire was designed by a panel of experienced trainer ophthalmologists in Kenya, South Africa and the UK. The questions and possible responses underwent an iterative process of refinement, through the participation of additional ophthalmology trainers in SSA. The web-based SurveyMonkey (San Mateo, CA, USA) platform was used for the questionnaire. The main groups of questions included current ophthalmology training; ophthalmology surgical training including simulation, perceptions of surgical training, surgical confidence, total numbers of surgeries performed and future career aspirations. The survey is available as *Extended data*.

### Participants and data collection

Eligible individuals for inclusion were all current ophthalmology trainees and recently qualified ophthalmologists (≤3 years since training completed), at any of the ophthalmology training institutions within the Eastern, Central and Southern Africa sub-regions ( Table 1). These doctors have synonymous titles in different sub-regions: trainee, registrar, or resident. For this study, the term ‘trainee’ is used.

A link to the web-based questionnaire was sent to all heads of eye departments and training programme directors of ophthalmology training institutions in the Eastern, Central and Southern Africa region, who forwarded to their trainees and recent graduates in September 2017. Three reminders were sent over a six-month period to those that had not completed the survey. The survey was also publicized via the International Agency for the Prevention of Blindness (IAPB) quarterly Africa Newsletter ^15^.

For closed questions, possible responses were on a five-point ordinal Likert scale: very satisfied, somewhat satisfied, neutral, somewhat dissatisfied, and very dissatisfied (see *Extended data*). Discrete numerical data was used for further quantitative questions. Free text responses were allowed for open questions. These were collated, and manual coding used before manual thematic analysis.

The total number of current ophthalmology trainees and ophthalmologists who completed training within the last three years was estimated at 240. To encourage attainment of responses, incentive strategies were employed. Participants were invited to enter a lottery for an iPad. Offering non-monetary incentives has been shown to increase survey responses by one half ^17^.

### Data analysis

Data were exported from SurveyMonkey into Excel (Version 15.31) for data management and analysis. For the quantitative data, we present descriptive statistics. Linear regression analysis was used for surgical experience and satisfaction with the training programme. For qualitative analysis, responses were collated from open question responses, and analysed thematically using manual coding. Verbatim quotations were used for common themes, and for comparison between different respondents.

## Results

### Respondent characteristics

Questionnaires were sent to 240 potential participants (140 current trainees and 100 recent graduates) and 124/240 (51.7%) responded. Assuming a population sample proportion of 50% (0.5), with a confidence level of 95%, the response rate of 52% (124/240) would mean a margin of error of 6.1% around the point proportion estimates, which we deemed acceptable.

The mean age of respondents was 30.8 years (range 26 – 46) and 58/124 (46.8%) were female. Respondents represented the full range of training years: 1 ^st^ Year, 28 (22.6%); 2 ^nd^ Year, 23 (18.5%); 3 ^rd^ Year, 17 (13.7%); 4 ^th^ Year, 13 (10.5%); and recent graduates, 43 (34.7%). Responses were received from all countries with training institutions, except Angola, Madagascar and Mozambique ( Table 1).

**Figure 1.**
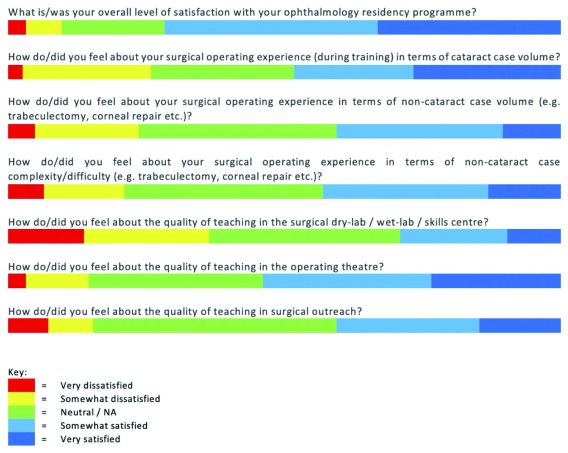
Horizontal bar diagram illustrating proportions of response on a five-point Likert scale.

In response to the question: ‘What is/was your overall level of satisfaction with your ophthalmology training programme?’, 89 (71.8%) were satisfied (combining very satisfied and somewhat satisfied), and 12 (9.7%) were dissatisfied (combining somewhat dissatisfied, and very dissatisfied) ( Figure 1).

### Surgical training

Participants were asked about live surgical training experience. Overall, satisfaction levels with surgical training experience were moderate: 67 (54.0%) were satisfied with the quality of base hospital operating theatre training, and 50 (40.7%) were satisfied with surgical outreach training. In total, 60 (48.4%) were satisfied with the cataract case volume during training. However, only 50 (40.7%) and 53 (43.1%) were satisfied with their non-cataract case volume and complexity, respectively ( Figure 1). A total of 92 (86.0%) stated that during training they use biometry on all cataract patients operated, however 4 respondents reported that this was not always available.

The numbers of procedures performed (during training) were reported ( Table 2). Of note, during the first two years of training, median cataract procedures performed was zero. For the 23 (60.5%) first and second year trainees who had performed no cataract surgeries, 10 (43.4%) were satisfied overall with the ophthalmology training programme, and 3 (13.0%) were dissatisfied. When specifically asked about cataract surgery case volume, 2 (8.7%) were satisfied, and 7 (30.4%) were dissatisfied. Conversely, for junior trainees who had performed over 10 cataract surgeries, 9 (69.2%) were satisfied overall with the training programme, and 7 (53.8%) were satisfied with cataract case volume. Linear regression analysis found a significant positive relationship between the numbers of cataract surgeries performed and overall satisfaction with the training programme (p=0.043).

**Table 2.** Total number of procedures performed by trainees (during training).

	Year 1/2 residents			Year 3/4 residents, Fellows			Graduates (past 3 years) *During training*		
Procedure	*Mean*	*Median*	*Range*	*Mean*	*Median*	*Range*	*Mean*	*Median*	*Range*
SICS	37	0	0-500	84	58	0-300	166	125	0-570
ECCE	28	0	0-300	64	10	0-400	51	2	0-300
Phaco	25	0	0-300	101	0	0-604	63	0	0-550
Paediatric cataract	1	0	0-15	3	0	0-20	8	2	0-100
Lid surgery	14	5	0-76	36	20	0-200	58	50	0-100
Lid surgery (trichiasis)	1	0	0-10	13	3	0-150	26	5	0-100
Evisceration	14	5	0-100	29	20	0-100	29	20	3-100
Exenteration	1	0	0-15	3	0	0-20	4	2	0-20
Corneal graft	0	0	0-0	2	0	0-23	1	0	0-5
Trabeculectomy	2	0	0-20	4	1	0-30	9	5	0-50
Strabismus (recti)	3	0	0-30	12	0	0-78	6	0	0-80
Retinal laser	30	0	0-300	144	15	0-900	85	28	0-422
Glaucoma laser	4	0	0-100	17	0	0-100	19	3	0-150

ECCE: Extra-capsular cataract extraction; SICS: Small-incision cataract surgery

**Figure 2.**
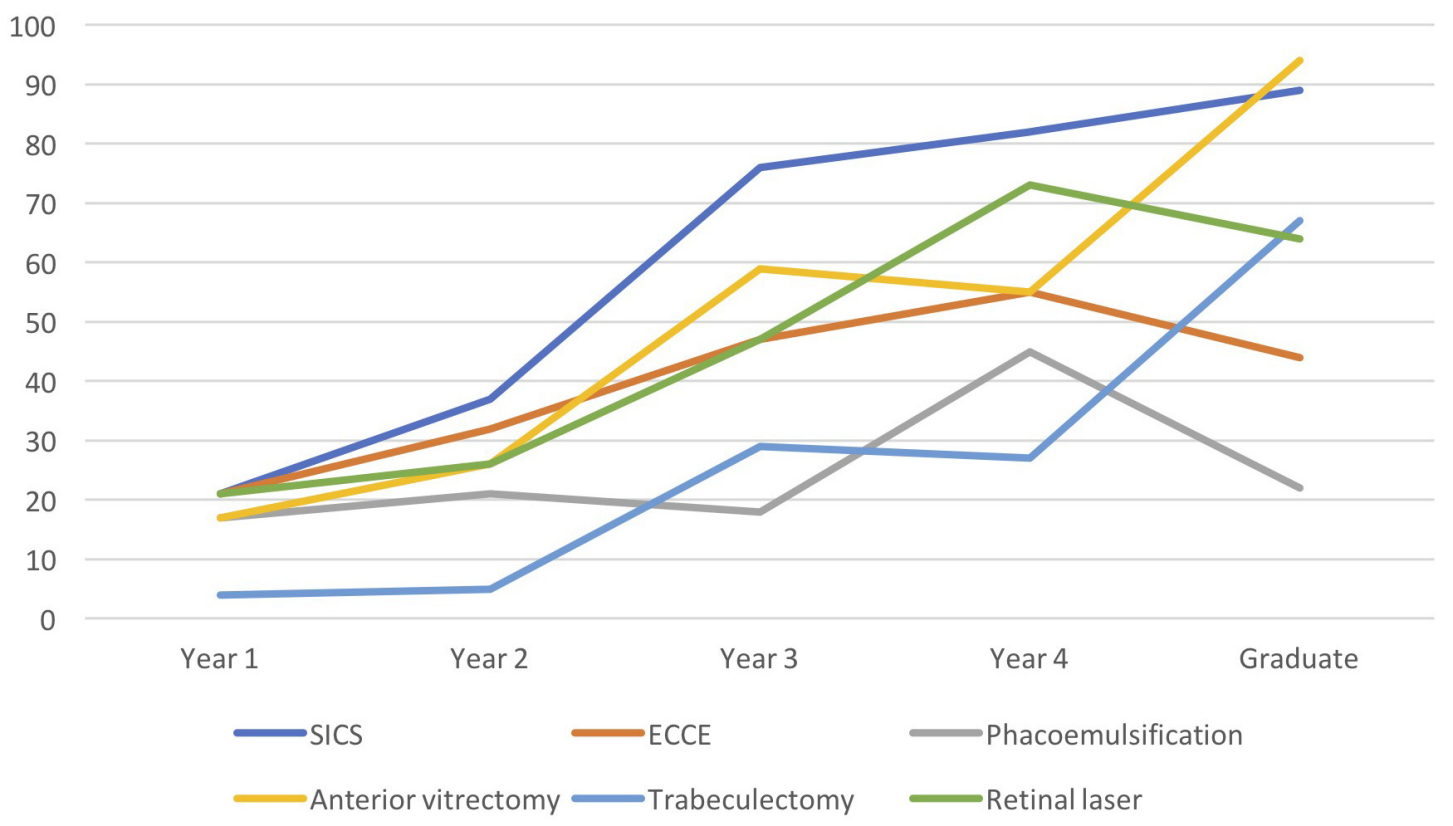
Surgical confidence percentages by year of training. Self-reported surgical confidence was assessed in response to the question “At the present time are you confident in performing the following types of procedure independently?”. The options were: ‘strongly agree’, ‘slightly agree’, ‘neutral’, ‘slightly disagree’, or ‘strongly disagree’. The graph plots the proportion responding ‘strongly agree’ or ‘slightly agree’ per procedure per year.

Participants were asked the question “At the present time, are you confident performing the following types of procedure independently?”, and offered a response on a five-point Likert scale. The degree of self-reported confidence increased steadily with increasing years of training ( Figure 2). Out of 28 senior trainees (year 3 or 4), the number agreeing or strongly agreeing that they felt confident in independently performing SICS was 22 (78.6%), extra-capsular cataract extraction 14 (50.5%), phacoemulsification 8 (28.6%), retinal laser 16 (57.1%), trabeculectomy 8 (28.6%) and lid surgery 15 (53.6%).

Thematic analysis of the qualitative responses regarding the ‘best surgical trainer’ found that the most commonly perceived positive attribute was patience (reported by 41 [41.4%]). This was followed by time afforded by the trainer (15 [15.2%]), skill/competence of the trainer (13 [13.1%]) and being calm (9 [9.1%]). One respondent described their best trainer as “patient, calm, and doesn’t take over too quickly”.

When asked about their ‘least good’ trainer, a similar theme arose: with ‘impatience’ being the most commonly reported negative attribute (reported by 24 [25.8%]). One trainee stated that the trainer was “not available, was in a hurry, was impatient. Did not even look at what I was doing”; and another that a trainer had “no time to supervise”. Trainee surgeons did not appreciate anger, with one describing “screaming and shouting”; and another how “shouting in theatre made the patient and I anxious”. When asked about areas for improvement, respondents mentioned supervision, development of wet-labs, increased surgical and clinical exposure.

Participants were asked “What is/was the part of your surgical training that you most feel needs improving?”. The most common areas identified were cataract (21 [22.6%]) and glaucoma (18 [19.4%]) surgery training. In total, 17 trainees (18.2%) highlighted the need for improved supervision, and 9 (9.7%) the need for better wet-labs/surgical skills centres. One trainee noted “I also wish we would be allowed to start surgery early enough based on our surgical skills and not on our year of study.”

### Simulation surgery training centres

Different terminology, such as surgical wet-lab, dry-lab and skills centre, is used to describe surgical training outside of the live operating theatre. For this study, the term ‘simulation surgery training centre’ (SSTC) encompasses all of these.

An SSTC was available for 92 (76.7%) trainees; however only 35 (29.2%) stated that there was a specific SSTC curriculum. One third (39 [33.6%]) had almost never spent time in SSTC during training, and only 24 (20.7%) had spent >2 hours per week. The median time spent in simulation training was 0–1 hours per week. Most (79 [71.2%]) stated they were ‘almost never’ supervised by a consultant in SSTC, and 5 (4.5%) stated they were supervised >50% of the time. Regarding supervision in SSTC by a fellow/senior trainee: half (59 [53.1%]) stated they were almost never supervised by a senior trainee, and 16 (14.4%) stated they were supervised >50% of the time. 

Less than half (52 [46.4%]) stated there were adequate consumables, 50 (44.6%) adequate instruments; only 25 (22.5%) had access to educational materials (books, videos, curricula). Regarding simulation eye materials available to residents, the most common were porcine (58 [52.7%]) and goat eyes (44 [40.0%]). Artificial eyes had been used for surgical training by 27 (24.5%), cow eyes by 26 (23.6%) and human cadaver eyes by four (3.6%). Only one trainee had experience with computerized/virtual reality simulation

### Cataract surgery outcome monitoring

Cataract surgical outcomes were routinely monitored by 88 (83.8%) trainees; this was mostly recording day one post-operative visual acuity. The most common reason for monitoring surgical outcomes was to ensure a successful outcome (26 [34.2%]), improve surgical skills (14 [18.4%]), learn from mistakes or complications (12 [15.8%]), for personal assessment (11 [14.5%]) and to build confidence (4 [5.3%]). Two surgeons mentioned ‘benchmarking’ against WHO standards. Four surgeons stated it was a requirement within the hospital.

For the challenges of monitoring outcomes, the most common issue was around follow-up difficulties and time constraints. One resident stated, “most cataract surgeries are done during community outreach and you hardly ever review the patients subsequently since we go back to our training centres”.

### Career aspirations and motivation

Participants were asked about their career intentions by sub-specialty. Although 41 (34.2%) expressed interest in general ophthalmology, the large majority (91 [74.0%]) also expressed interest in one or more sub-specialty areas ( Table 3). A total of 22 (18.3%) indicated an interest in academia or research.

**Table 3.** Future career preferences.

Ophthalmology career intentions: “ *At this time, what is your career* *choice?” (select one, or more which apply)*		
Specialty/Clinical area	n/120	%
General Ophthalmology	41	34.2
Cataract	28	23.3
Vitreo-retinal	27	22.5
Oculoplastics	21	17.5
Community Eye Health	20	16.7
Glaucoma	20	16.7
Cornea	19	15.8
Paediatric and Strabismus	19	15.8
Medical Retina	11	9.2
Future work environment preferences: *“What kind of eye unit do* *you plan to mostly work in (more than 50% of time)?”*		
**Type of health facility**	**n/120**	**%**
Government hospital	41	34.2
University Teaching Hospital	34	28.3
Private Practice	27	22.5
Mission Hospital	10	8.3
Community / Public Health	7	5.8
Academia (non-clinical)	1	0.8
Geographic working preference: “ *Geographically, where would* *you plan to work (select all that apply)?”*		
**Future work geography**	**n/121**	**%**
Home country	93	76.9
Abroad	14	11.6
Capital city	27	22.3
Other urban/city	31	25.6
Rural area	14	11.6

Footnote: Some of the respondents did not answer all questions; the denominator is provided for each.

Over one-third of respondents (41 [34.2%]) stated a future work-place preference for a ‘Government Hospital’. The second most common was University Teaching Hospital (34 [28.3%]) ( Table 3). Although private practice (27 [22.5%]) was third; many did identify a balance, with two residents stating, “private practice is more profitable than working in government, but the latter is more satisfying” and “mixed public and private because public is difficult to organize but important for poor people”.

We asked about the country they intended to practice in long-term: 93 (86.9%) plan to work in their home country, while only one in eight (14 [13.1%]) stated a desire to work abroad. Totally, 58 (77.3%) specifically stated they would want to work in either the capital city or other urban environment, whereas only 15 (20.0%) stated they would plan to work in a rural area. When asked their reasons for their choice of where to work, 39 (32.2%) stated that this was where the need was. Family also plays a large part in the decision, for 21 (17.4%) family was the main reason, with one respondent explaining: “whereas to work in a rural area is serving the most in need population, the minimum social life is not adequate for my family. Any urban area is an option since I can still reach out [to] many needy people and get a favourable environment for my family”. Further thematic analysis of the reasons given for this work location choice included quality of life, pay and private practice.

## Discussion

This is the first regional survey of ophthalmologists in training in Eastern, Central, and Southern Africa. We did not directly compare training institutions, but rather provide institutions as a current benchmark for the region. If a repeat survey is undertaken using the same methodology in a few years this will provide a reference point for comparison.

Emphasis has been given in this current study to trainee experience, satisfaction and confidence. Competence is challenging to measure in surgical training. This is in part due to the lack of validated assessment tools and benchmarks, but also variability in training programme format. Contemporary approaches to training and traditional Halstedian apprentice-style surgical training is being replaced by more outcome-defined, demonstrable, learner-centred, assessable curricula of competencies ^18^. Competency-based ophthalmology curricula and tools have been developed. These include the ophthalmology milestones project ^19^, and workplace-based assessments ^20^, however CBME curricula and assessment tools are not yet uniformly used throughout SSA ^21, 22^.

The overall level of satisfaction with ophthalmology training programs was fair, with seven out of ten satisfied, and less than one in ten dissatisfied. This is encouraging, as much has been achieved in the past years despite the challenge of enrolment numbers increasing. Great efforts have been made to develop regional standards of ophthalmic training, develop standardized curricula and post-graduate fellowship or board level-exams. This is less than a recent survey of residents in the USA which reported a 94% ‘highly satisfied’ rating ^8^.

However, when focusing on surgical education, only 57% were satisfied with their cataract case volume in training; and this reduced to 20% for first and second year trainees. The median number of cataract surgeries performed by first and second year trainees was zero. This is a cause for concern, considering that some training programs are only three years in duration. For all cataract surgeries combined, the total median and mean numbers performed by senior trainees were 96 and 222, respectively. This reflects the large range (3 to 1,100) and the variation in the cataract surgical procedures taught in SSA. A large survey in the USA showed the median and mean number of cataract procedures completed by trainees by the end of training was 100 and 113, respectively ^23^. A more recent survey of US program directors showed that third-year trainees had completed a mean of 155 phaco cataract surgeries as primary surgeon ^24^. These figures are more almost double the 83 minimum average number of cataract operations that a training program must provide to gain accreditation ^25^.

Senior (final) year trainees are under great pressure to reach these surgical numbers and have their surgical logbooks completed. This often leads to a hierarchical approach to surgical training and opportunity. Final year trainees often have complete priority over junior trainees for cataract surgical training cases. For senior trainees and recent graduates in this survey, cataract experience is reasonable with nearly half having completed >100 procedures during training. This may not be a high enough number ahead of being sent out to independently run a service in a remote setting.

The amount of retinal laser experience was good, and this is encouraging considering recent increases in the incidence of diabetic retinopathy across the continent. Paediatric cataract and corneal graft surgery can certainly be within the domain of sub-specialist fellowship-level training, and therefore surgical experience in this survey may be acceptable. However, glaucoma is the third most common cause of blindness globally ^26^, and it is of concern that mean and median trabeculectomies performed by senior trainees and recent graduates is less than ten. Non-glaucoma-specialist ophthalmologists often shy away from performing a surgical trabeculectomy, as patient satisfaction is low: vision will often be worse post-operatively, never better ^27, 28^. Surgical management of advanced glaucoma is often the first line of treatment and should be within the remit of a general ophthalmologist.

The surgical experience of trainees in evisceration/enucleation is high. The mean and median number of procedures performed by senior trainees and graduates (during training) was twenty or more. This can be explained by the prevalence of severe infections, trauma, and tumours.

It is often a challenge within ophthalmology training institutions, sometimes oversubscribed, for surgical trainers to have enough time and appropriate cases to teach surgery effectively. Exploration of innovative and effective ways to train efficiently and safely. To this end, some training institutions have established working relationships with other eye units as satellite surgical training centres.

Although SSTCs were available for over two-thirds of residents, less than one-third were satisfied with the quality of teaching provided, and 40% had almost never spent time in an SSTC. This is in line with trainee ophthalmologists’ perspectives in Nigeria where only 33.3% had supervised SSTC sessions ^29^. Ophthalmic simulation-based surgical education is underutilized and unstructured. Yet it offers the potential to substantially enhance the quality, speed and safety of surgical skills acquisition ^30^. For simulation-based surgical education to work, instruction, supervision, feedback, a curriculum, and outcome (surgical competency) measurement are required ^31^. 

The culture of the training environment is also critical. Trainees’ appreciation of patience, and not anger or impatience, is in line with surgical education throughout the world ^32, 33^; but may be more so in Africa. As Thomas Fasokun, Professor of Adult Education at Ile-Ife (Nigeria) concludes: “anxiety is one issue that dominates the participation of African adults in learning. For learning to take place, the level of anxiety in the learner must be minimized. Adults are quick to react unfavourably to unnecessary pressure on them” ^34^. Adult-orientated teaching methods need to be employed in ophthalmic surgical education.

Recently ‘training-of-trainers’ initiatives have been successfully implemented in the COECSA region in partnership with the International Council of Ophthalmology (ICO) and The Royal College of Ophthalmologists, UK. This training in teaching and assessment skills has had a positive impact on training ^35, 36^.

The high pressure and stressful environment of the operating theatre, the huge burden of disease, and the need for a calm environment to learn and practice surgery naturally lean towards a simulation surgical skills centre as a potentially valuable solution. Furthermore, we could use educational theory underpinned simulation-based surgical education to provide calm and high-impact training. Trainees could be ‘competent’ in cataract or glaucoma surgery using simulation, to then be afforded opportunities for supervised surgical training sooner in their training program.

SSA has an age-standardized prevalence of blindness of 1.3%, 35% of which is due to cataract; and 2.7 ophthalmologists per million population. Taking a cataract surgical rate of 500 as typical for SSA, this means the average ophthalmologist in SSA currently does just 185 cataracts per year (500/2.7). The share of these cases used for training is not high as government training institutions are not typically high-volume. Funding for ophthalmology training, including surgical education, is a challenge not only in many countries in SSA, but worldwide. Some of the money brought in for training needs to be spent on increasing cataract surgical case numbers so the substrate for training exists. Some training institutions are now looking towards developing stronger referral systems rather than an expansion of outreach, to improve patient flow.

Career preferences for surgical sub-specialities (cataract, vitreo-retinal, cornea and oculo-plastics) is in line with other surveys around the world. It is encouraging that the most common career aspiration was ‘general ophthalmology’ (34%). This is consistent with a recent small cross-sectional study of ophthalmology masters students in Eastern Africa, where 69% of respondents wanted to sub-specialise ^37^. With 2,700 ophthalmologists for one billion population in SSA, most ophthalmologists do indeed need to be generalists. Sub-specialty career preferences do not necessarily align with training opportunities and service needs. There is ongoing collaborative discussion in the region as to how sub-specialists would be trained, and on what needs basis. Universal eye health will only be achieved if difficult and challenging sub-specialized needs are met as well as cataract and URE. There is a need for collaborative work-force planning upon which to base the need for sub-specialist ophthalmology training. However, such efforts should be cognizant of the ongoing need to tackle the burden of blindness and vision impairment due to cataract and URE.

Most (77%) respondents plan to work in their home country. However, only one in ten stated they would plan to work in a rural area. A recent study of human resources for eye health in 21 countries in SSA found that 67.2% of ophthalmologists work in the national capital cities ^38^. The ophthalmologist plays an important part in the eye care team. There are currently too few ophthalmologists to meet the current need, however the situation should gradually improve over the next decade. The emphasis should not necessarily be on placing ophthalmologists in rural areas with low population density. With ophthalmologists predominantly in larger towns and national capital cities, there is a greater need for task-shifting; better integration of primary, secondary and tertiary health care; and strengthening of referral systems. 

The third most common workplace preference was private practice (22.5%). With a quarter of ophthalmologists stating a desire to serve and help the community in need, and nearly a quarter planning for private practice; it is interesting that these were not mutually exclusive. As one participant illustrated “I want to serve the underserved communities in the rural areas and at the same time run a private practice in the city”. Another participant further added “There is a better fiscal opportunity, but also it is easier to get donor funds for research or outreach”. Dual practice is common among health professionals worldwide, however further research is necessary to ascertain the impact on the achievement of universal health coverage ^39^.

## Conclusion

This survey illustrates that although the mean number of cataract surgeries performed (during training) by senior trainees or recent graduates is adequate, the vast majority of these are performed in the final years of training. More than half of junior trainees’ cataract experience is zero. Is this the best approach to producing proficient ophthalmic surgeons: to cram surgical numbers in the final year, rather than facilitate gradual and sustained building of competence and confidence?

This survey allows us to collectively raise research questions regarding training. These may include exploring qualitatively why some trainees get so few surgical cases, and quantitatively to investigate the link between low surgical numbers in training and complication rates in the first two years of consultant work. The results will inform us as we continue discussions about minimum standards.

Ophthalmic surgical education and opportunity is complex. It would perhaps make sense to train trainee eye surgeons to a level of competence rapidly and safely using simulation; then immediately follow-up with live supervised surgical training. This approach would accelerate training, improve trainee competence, confidence and satisfaction in training; and ultimately improve outcomes and services for patients.

## Ethics approval and consent to participate

This study was approved by the research ethics committees of the London School of Hygiene & Tropical Medicine (11795) and the University of Cape Town (259/2017). Participants were provided with information about the nature of the survey prior to participation. Participation was entirely voluntary. As the survey was anonymous, individual written consent was not required prior to voluntary participation.

## Data availability

### Underlying data

LSHTM Data Compass: Survey of Ophthalmologists-in-Training in Eastern, Central and Southern Africa - Survey data and questionnaire, https://doi.org/10.17037/DATA.00001464 ^40^.

This project contains the following underlying data:

- Trainee_Survey_SSA_-_Complete_Responses_Anonymised

This data is under restricted access due to the assurance given to participants that responses would be kept completely confidential. Specifically, some training institutions and even countries may only have one or two trainees who could therefore easily be identified by default. The data set can be accessed by completing the Request Form, which requires that the intended use for the data is specified. Data available under the LSHTM Data Compass Data Sharing Agreement.

### Extended data

LSHTM Data Compass: Survey of Ophthalmologists-in-Training in Eastern, Central and Southern Africa - Survey data and questionnaire, https://doi.org/10.17037/DATA.00001464 ^40^.

This project contains the following extended data:

- Questionnaire
- Data codebook

Data available under the LSHTM Data Compass Data Sharing Agreement.
