# Supplementary material for: Survey of ophthalmologists-in-training in Eastern, Central and Southern Africa: A regional focus on ophthalmic surgical education
Source: Wellcome Open Res. 2019 Nov 27;4:187. [Version 1] doi: 10.12688/wellcomeopenres.15580.1 (PMC6913214; doi:10.12688/wellcomeopenres.15580.1)
Supplement: Peer review history

# Peer review: Reviewer response for version 1

**DOI:** 10.21956/wellcomeopenres.17061.r37179

**Published:** 2019-12-11

**Authors:** Brad Hal Feldman

**Affiliations:** 1 Cornea Service, Department of Ophthalmology, Jefferson Medical College, Wills Eye Hospital, Philadelphia, PA, USA; 2 Vantage EyeCare Philadelphia Eye Associates Division, Philadelphia, PA, USA

This is an important report on an area that has not been well studied. I applaud the authors for taking on this project. The article is overall well written and clear. 

 I have some questions about the data. In Table 1 there are 119 trainees spots listed per year, yet the authors assume that only 240 would have completed training in the past 3 years. This might be explained a number of ways (I know some programs are new), but needs explanation. We need an explanation as to how the assumption was made of 240 total invited participants. My calculation would be at least double that number. This significantly affects our interpretation of the generalizability of the data across SSA.

 The data is also quite skewed by the fact that just 4 of the 24 programs represent about half of the respondents to the survey. Because of this, presenting median and mean may not be as helpful as other graphic displays of the range of responses. The mere fact that there is so much variability is extremely important and could be better conveyed to readers. 

 I see some errors on the table. Please have the authors double check the names of the Institutions. For instance, it is the Rwandan International Institute of Ophthalmology, not the shorter name given. 

 Also, they state responses were received from all but a few countries, but that does not match what is represented in Table 1. Please double check the text and tables. 

 Asking about surgical experience qualitatively is important, but it brings into question the expectations for performance set by each program or in the mind of the respondent. If a program accepts a 15% complication rate, than trainees may feel very satisfied that they can achieve this. It may be better to give the survey participants data about benchmarks for surgical outcomes (say 1% complication rates) and ask how well prepared they feel they'll be to meet those benchmarks. Currently, the questions are extremely subjective even as qualitative measures.

 Likewise, while gathering surgical numbers is important, we need more information about the quality of those cases. Trainees were asked about "primary" cases but not given a definition of this - were all interpreting or understanding this the same? How many cases were supervised by an attending, by senior residents or at all? Quality is a huge factor that many times is as or more important than quantity. 

 The article mentioned curricular guidelines created by some SSA organizations such as COECSA. If these contain any surgical guidance or minimums, it would be helpful to have that information to put the surgical numbers of trainees in perspective. 

 Likewise, to compare cataract surgery numbers in SSA to the USA is troublesome. The surgical technique is entirely different with a different learning curve and complication curve (see published literature on this). It would be better to compare the SICS numbers to those of training programs in India. I'd consider adding this information to the paper if available.

I confirm that I have read this submission and believe that I have an appropriate level of expertise to confirm that it is of an acceptable scientific standard.

# Peer review: Reviewer response for version 1

**DOI:** 10.21956/wellcomeopenres.17061.r37181

**Published:** 2019-12-02

**Authors:** Mohammed Mahdi Abdull

**Affiliations:** 1 Department of Ophthalmology, Abubakar Tafawa Balewa University Teaching Hospital, Bauchi, Nigeria

This is a very significant study. The authors conduct the first large-scale survey in in the African sub-region covered. The introduction clearly cites all the relevant studies especially those conducted in other parts of Africa. The methodology employed appears to be the best way to get the necessary responses from the study participants. A response rate of just over 50% is quite low, but considering the time frame given, the incentive by lottery and reminders perhaps that is the best that could be obtained. The results are clearly presented with the tables easy to understand. The presentation of the responses to the Likert scale on the horizontal bars gives a visual appreciation of the responses. The survey raises the question why junior trainees have almost no cataract surgery experience, would this have been different if the response rate was higher. The conclusions drawn from the survey are valid and more studies are needed to explore the raised issues.

I confirm that I have read this submission and believe that I have an appropriate level of expertise to confirm that it is of an acceptable scientific standard.
